# Disability weights from a household survey in a low socio-economic setting: how does it compare to the global burden of disease 2010 study?

**DOI:** 10.3402/gha.v9.31754

**Published:** 2016-08-17

**Authors:** Ian Neethling, Jennifer Jelsma, Lebogang Ramma, Helen Schneider, Debbie Bradshaw

**Affiliations:** 1 Burden of Disease Research Unit, South African Medical Research Council, Cape Town, South Africa; 2 Department of Health and Rehabilitation Sciences, University of Cape Town, Cape Town, South Africa; 3 School of Public Health, University of the Western Cape, Cape Town, South Africa

**Keywords:** disability weights, health state preferences, disability adjusted life years, DALY, South Africa, pairwise comparisons

## Abstract

**Background:**

The global burden of disease (GBD) 2010 study used a universal set of disability weights to estimate disability adjusted life years (DALYs) by country. However, it is not clear whether these weights can be applied universally in calculating DALYs to inform local decision-making. This study derived disability weights for a resource-constrained community in Cape Town, South Africa, and interrogated whether the GBD 2010 disability weights necessarily represent the preferences of economically disadvantaged communities.

**Design:**

A household survey was conducted in Lavender Hill, Cape Town, to assess the health state preferences of the general public. The responses from a paired comparison valuation method were assessed using a probit regression. The probit coefficients were anchored onto the 0 to 1 disability weight scale by running a lowess regression on the GBD 2010 disability weights and interpolating the coefficients between the upper and lower limit of the smoothed disability weights.

**Results:**

Heroin and opioid dependence had the highest disability weight of 0.630, whereas intellectual disability had the lowest (0.040). Untreated injuries ranked higher than severe mental disorders. There were some counterintuitive results, such as moderate (15th) and severe vision impairment (16th) ranking higher than blindness (20th). A moderate correlation between the disability weights of the local study and those of the GBD 2010 study was observed (*R*^2^=0.440, *p*<0.05). This indicates that there was a relationship, although some conditions, such as untreated fracture of the radius or ulna, showed large variability in disability weights (0.488 in local study and 0.043 in GBD 2010).

**Conclusions:**

Respondents seemed to value physical mobility higher than cognitive functioning, which is in contrast to the GBD 2010 study. This study shows that not all health state preferences are universal. Studies estimating DALYs need to derive local disability weights using methods that are less cognitively demanding for respondents.

## Introduction

Disability weights are used to quantify time of healthy life lost due to disability and represent the severity of health loss on a scale of 0 (full health) to 1 (death) (1). They are used to calculate the morbidity component of disability adjusted life years (DALYs), which allows mortality and morbidity outcomes to be combined into a single metric, allowing for a comprehensive assessment of health conditions. The DALY was first introduced in the 1990s and has been used by the global burden of disease (GBD) group to estimate global population health ever since (1). Disability weights are based on valuations of health states, which are short, lay descriptions of health with no accompanying disease label; they can encompass the effects of several diseases, for instance, blindness can be the effect of diseases such as diabetes and stroke.

In order to derive disability weights, it is necessary to define the group whose responses should be elicited, the method of health state valuation measurement, and the health state descriptions (2). Study participants consist of either health experts, patients experiencing the health effects, caregivers, or the general public (3). There are various methods used to elicit health state responses. Some of these require study participants to make trade-offs either in time (TTO) or person-years (PTO). Another method is the standard gamble approach, which requires specifying a risk of death against improvement in health, and other options are the pairwise trade-off and willingness to pay valuation methods, which involve ranking health states against each other (2, 4). A health state description can describe a disease condition in generic or disease-specific terms (2). The various methodological options used to derive disability weights could result in differing estimates for the same health states and thus influence the overall burden of disease estimates for specific disease conditions (2).

In 1996, the GBD study constructed disability weights based on the valuation of different health states by health experts (1). These were used as a universal set of disability weights in the 1996 GBD DALY and also in subsequent GBD estimates (5, 6).

The 1996 study as well as subsequent GBD studies have been criticised for excluding the valuation of health states by the broader community and therefore not reflecting a global understanding of health (7). Studies have indicated that the weights are not universal. For instance, Jelsma et al. (8) compared the ranking of the 22 indicator conditions used in the 1996 GBD between health professionals and non-professionals. They reported that the rankings of the indicator conditions of Zimbabwean health professionals were very similar to those of the GBD (Spearman's *ρ*=0.912) but that the correlation between health professionals and the lay public was much lower (*ρ*=0.341). They concluded that the weights represented the values of a small group of educated elite, rather than those of society as a whole. Ustün et al. (9) compared the ranking of 17 health conditions from 241 informants (health professionals, policy makers, people with disabilities, and their carers) across 14 countries. They found significant differences (*p*<0.05) in rankings between countries for 13 of the 17 health conditions, whereas 5 health conditions were ranked significantly different (*p*<0.05) between the different informant groups. The findings of these studies indicate that the disability weights derived by the 1996 GBD study may not be universally applicable.

The GBD 2010 study sought to address this critique by undertaking a large-scale re-estimation of disability weights. The researchers assessed health state preferences of 220 unique health states of the general public through population-based surveys (10). A household survey was undertaken in five countries (Bangladesh, Indonesia, Peru, Tanzania, and the United States) as well as a web-based survey to elicit responses from populations diverse in language, culture, and socio-economic status (10). Respondents in the household surveys were given paired comparison questions of health states whereas the respondents in the web survey were assigned to one of four different survey versions, which included paired comparison and population health equivalence questions. To assess country differences in health state valuations, paired comparison health state valuations were combined from all the pooled analyses and compared to each household survey. A high degree of consistency in ranking was found between sites (*r*=0.90 or higher) for all except Bangladesh (*r*=0.75). Based on this finding, the authors disputed the hypothesis that health valuations vary widely across cultural, educational, and environmental circumstances.

Although the GBD 2010 study was intended to assess a diversity of respondents, 57% had tertiary education and 17% had only primary education or less. In addition, more than 50% of the respondents participated in the web survey of which 93% had tertiary education and might have had prior experience with the GBD approach because of the type of recruitment used to attract the web participants. This suggests that the study participants may not have been as diverse as intended and that the five selected sites were perhaps not sufficient to represent the global population.

The South African team approached the GBD researchers because they wished to replicate the GBD study in the small suburb of Lavender Hill in Cape Town. Local researchers were trained by the GBD team and were allowed to utilise the GBD methodology and data collection instruments. However, as the South African sample was not part of the original global sample and the sample size was much smaller, data from the Cape Town sample were not included in the overall GBD 2010 analysis.

The GBD improved its methods in deriving disability weights for its 2010 study, but has not been able to refute all criticisms related to the universality of the weights (11, 12). There is still a need to interrogate to what extent the weights represent the preferences of disadvantaged and less-educated communities in particular. The existence of data collected using almost identical methodology to the main study allowed us to compare the global health state preferences with those representing the resource-deprived community of Lavender Hill in Cape Town.

The GBD study assumes that health preferences are generalisable across different populations despite differences in socio-economic status, and cultural and political beliefs (10). This hypothesis was explored by comparing the disability weights of this study to those in the GBD 2010 study.

## Methods

As explained here, the methodological design and tools used in this study were conceptualised by researchers of the GBD 2010 study, led by Joshua Solomon (10). The South African data collection team was trained by the GBD researchers, and the software developed by the GBD team was used to gather the local data. The method was similar to that used in the GBD 2010 study with the following important exceptions. Firstly the local study used the TTO valuation method to assess the indicator conditions whereas the GBD 2010 study used a population health equivalence method. The local study assessed 51 health states compared with 220 health states in the GBD 2010 study. In addition, the local study used household personal interviews to elicit responses, whereas the GBD 2010 study used household personal interviews as well as telephonic interviews and a web survey method.

### Study design and research setting

A household survey was conducted in the resource-deprived community of Lavender Hill, Cape Town, whose residents are bilingual in English and Afrikaans. Census data indicate that this suburb has an approximate population of 32,000, with 19% of people aged 20 years and older having completed Grade 12 or higher, 58% of the labour force being employed, and 59% of households having a monthly income of R3,200 (~US $228) or less (13). The dwellings consist of small apartments, houses, and informal settlements each representing about one-third of all dwellings in the area.

### Sampling

An aerial map was used to divide each dwelling type into approximately equal numbers of clusters from which two clusters were selected for each type. Four streets were randomly selected from each cluster and every third dwelling in the street was visited until 20 eligible adults were found. An adult aged 18 years and older was randomly chosen from each household using a statistical package for the social sciences (SPSS) computer algorithm, designed by the GBD team, after obtaining information on the sex and age of each household member.

### Sample size calculation

A sample of 700 respondents and 51 health states were assessed to have a margin of error not higher than 0.7 at the 95% confidence interval by simulating the mean relative error against a benchmark of 2,500 respondents and 100 health states.

### Data collection procedures and instruments

Data collection occurred between September 2009 and March 2010. Face-to-face interviews were conducted in English by trained interviewers and facilitated by a computer-assisted personal interview programme (CAPI) created by the GBD researchers, with survey questions and response options displayed one at a time in the appropriate order on a laptop screen. Participants did not receive any remuneration for their participation. Interviews lasted an average of 19 min and 24 sec, whereas it took 8 min and 24 sec on average for all the pairwise comparison questions, and 1 min and 12 sec for the TTO valuation.

The CAPI programme was used to randomly choose the different health state valuation questions. It also contained questions on demographics, individual and household assets, marital status, and education.

The health states were presented to respondents as brief lay descriptions, using non-clinical vocabulary that highlighted the symptoms and functional consequences of each health state. These descriptions were the same as those used in the GBD 2010 study (10). Two health state valuation techniques were used to assess health state preferences. Firstly, in a pairwise health state trade-off, respondents were presented with two descriptions of hypothetical people each with a different health state and asked which they thought was the healthiest. Each respondent completed 15 pairwise comparisons randomly selected from a possible 51 health states, which were extracted from a list of 107. Secondly, a TTO health valuation was used to assess the 10 health states used as indicator conditions with each respondent required to valuate one indicator condition. In a TTO, respondents are asked to choose between living 10 years with a health state with some mental or physical limitation, and living a shorter period without any limitation. Because hypothetical scenarios were used in both the pairwise trade-off and TTO valuation techniques, no sensitive information was collected. The fieldworkers recorded all answers on the SPSS computer program, and all data were consolidated on a central server.

### Pilot study

The questionnaire was piloted in the study population prior to the commencement of data collection to assess whether respondents were able to understand the questions. In addition, a test was done to assess the cognitive ability of the study participants. It was found that the methodology was feasible.

### Ethical considerations

The study was approved by the Health Research Ethics Committee of the University of Cape Town. Informed consent was obtained from all participants.

### Analysis

Analysis was performed using Stata/IC 12.0 and Microsoft Excel 2010. Descriptive analysis was undertaken for the variables sex, age, marital status, and education. Marital status distinguished between those currently married, those who had been married and were divorced, widowed or separated, and those who had never been married. Divorced implied a legal separation, whereas separated implied living apart without a legal process having been followed. Living together implied living as man and wife without having gone through the process of legal marriage. Education distinguished between the different levels of schooling, that is, primary, secondary, higher education, and no schooling. Some primary or some secondary refers to respondents who had attended primary or secondary school without completing the highest grade.

Disability weights were derived for the 10 health states used as indicator conditions. This was done by deriving the disutility (1-year/10) of the upper and lower limit of each health state. The disutilities were then logit transformed, as was done in the GBD 2010 study, because it allows for normally distributed error (10). The logit-transformed disabilities were then used to conduct an interval regression. Respondents were given the option of choosing between living 3, 5, or 7 years in perfect health instead of 10 years with a particular health state. However, as there was a possibility of respondents choosing values in between those presented to them, an interval regression was used. The resulting coefficients were then back transformed onto the 0 to 1 disability weight scale.

To analyse the paired comparison valuations, a probit regression was used to assess the relative difference in severity of health states, following the GBD 2010 approach (10).

Rescaling of the probit coefficients was assessed using two methods. Firstly, a linear regression against the logit-transformed disability weights of the indicator conditions was run, with the resulting slope and intercept used to transform the coefficients onto the disability weight scale. Secondly, a lowess regression of the probit regression coefficients against the logit-transformed disability weights of the GBD 2010 (10) study was run, following the method used in the study by Haagsma et al. (14). The predicted smooth coefficients were then back transformed to yield disability weights onto the 0 to 1 scale. The probit coefficients were then linearly interpolated between the upper and lower limit of the disability weights.

These disability weights were compared with the disability weights of the GBD 2010 study, using a Pearson's correlation coefficient with significance set at *p*<0.05.

A 95% confidence interval for each disability weight was derived by lowess regression of the upper and lower limit of each probit regression coefficient, with the upper and lower logit-transformed GBD 2010 disability weights.

## Results

Of the 741 people selected for interviews, 62 refused to answer any questions and 2 partially completed the health valuation questions. The results are therefore based on the answers of the 677 respondents who completed all the questions. The age range of the respondents was between 18 and 81 years, with an average age of 46 years; 59% were female; 94% were between the economically active ages of 18 and 65 years; 35% were married; 21% had completed Grade 12; and, of these, 3% had studied further (Table 1).

**Table 1:** Background characteristics of the sample (*N*=677)

Characteristics	*N* (%)
Sex	
Female	397 (58.6)
Male	280 (41.4)
Age category	
18–29	227 (33.5)
30–39	127 (18.8)
40–49	110 (16.2)
50–59	115 (17.0)
60+	98 (14.5)
Marital status	
Never married	263 (38.8)
Currently married	240 (35.4)
Separated	20 (2.9)
Divorced	42 (6.2)
Widowed	82 (12.1)
Living together	30 (4.4)
Education	
No schooling	4 (0.6)
Some primary	92 (13.6)
Completed primary	65 (9.6)
Some secondary	353 (52.1)
Grade 12	140 (20.7)
Higher	23 (3.4)

On average, respondents chose spinal injury at the neck level as the most severe condition with a disability weight of 0.700 using the TTO valuation method, whereas moderate angina pectoris had the lowest disability weight of 0.029 (Table 2). The indicator conditions were not used for rescaling because they showed a negative correlation with the probit coefficients, which results in disability weights that are inversely related to the severity of health states as indicated by the probit coefficients. The lowess approach was therefore used as an alternative rescaling procedure.

**Table 2:** Average willingness to trade-off time and ranking of indicator conditions by disability weight

Rank	Health state	Average time in years willing to trade-off	Disability weight	95% CI LL	95% CI UL	SE	Coefficient of variation	*N*
1	Spinal cord lesion at neck, treated	5.13	0.700	0.450	0.869	0.09	12.3	67
2	Dementia, moderate	5.05	0.666	0.422	0.845	0.09	13.7	76
3	Major depressive disorder, moderate episode	4.49	0.431	0.215	0.677	0.13	29.1	83
4	Neck pain, acute, severe	4.19	0.362	0.162	0.624	0.13	37.0	63
5	Anaemia, severe	4.06	0.290	0.111	0.574	0.14	49.8	63
6	Distance vision, severe impairment	3.39	0.170	0.058	0.407	0.12	71.0	56
7	Musculoskeletal problems, legs, moderate	3.04	0.092	0.028	0.260	0.09	93.9	54
8	Amputation of one leg, long term, without treatment	2.38	0.052	0.020	0.125	0.04	72.2	70
9	Hearing loss, moderate	2.37	0.038	0.015	0.091	0.03	72.0	82
10	Angina pectoris, moderate	2.21	0.029	0.010	0.080	0.03	88.2	63

CI: confidence interval, LL: lower limit, UL: upper limit, SE: standard error, N: number.

Table 3 displays the disability weights of the health states used in the pairwise comparison valuation method by health state domains. Heroin and opioid dependence had the highest disability weight of 0.630, followed by severe brain injury (disability weight 0.536), whereas severe intellectual disability ranked lowest (disability weight 0.04). Untreated injuries such as amputation of one leg (disability weight 0.504) and fracture of the radius or ulna (disability weight 0.488) ranked high at third and fourth, respectively. The health states with the highest disability weights seem plausible, as they have higher severity. Similarly, health states with milder severity such as primary (disability weight 0.047) and secondary infertility (disability weight 0.108) and periodontitis (disability weight 0.175) ranked low at 50th, 48th, and 46th, respectively. However, some of the results seem counterintuitive such as severe and moderate vision impairment ranking higher than blindness. Respondents chose the first health state mentioned in a paired comparison as the healthier option 53% of the time, while the first health state was also selected 53% of the time when respondents were asked to decide between health states with similar severity.

**Table 3:** Disability weights and rank for health states by health state domain

Health state	Rank	Local disability weights	95% CI LL	95% CI UL
Infectious disease				
HIV: symptomatic, pre-AIDS	9	0.417	0.285	0.502
Tuberculosis without HIV infection	18	0.368	0.260	0.430
Ear pain	5	0.486	0.318	0.606
Cancer				
Terminal phase with medication (for cancers, end-stage kidney or liver disease)	12	0.407	0.258	0.526
Cardiovascular and circulatory disease				
Angina pectoris, severe	41	0.240	0.159	0.304
Angina pectoris, moderate	42	0.222	0.151	0.275
Diabetes, digestive and genitourinary disease				
Infertility, primary	50	0.047	0.015	0.100
Infertility, secondary	48	0.108	0.060	0.166
Neurological disorders				
Dementia, severe	7	0.475	0.315	0.586
Dementia, moderate	37	0.258	0.168	0.331
Dementia, mild	32	0.301	0.185	0.402
Parkinson's disease, moderate	14	0.398	0.260	0.500
Mental, behavioural, and substance-use disorders				
Schizophrenia, acute state	8	0.465	0.303	0.583
Major depressive disorder, severe episode	24	0.344	0.224	0.436
Major depressive disorder, moderate episode	35	0.270	0.192	0.317
Heroin and other opioid dependence, moderate to severe	1	0.630	0.419	0.770
Alcohol-use disorder, severe	23	0.348	0.225	0.443
Alcohol-use disorder, moderate	19	0.364	0.235	0.463
Alcohol-use disorder, mild	47	0.152	0.104	0.194
Cannabis dependence	34	0.283	0.187	0.357
Autism	10	0.413	0.271	0.516
Intellectual disability, severe	51	0.040	0.019	0.078
Borderline intellectual disorder	31	0.304	0.201	0.381
Hearing and vision loss				
Distance vision, blindness	20	0.361	0.228	0.468
Distance vision, severe impairment	16	0.374	0.247	0.466
Distance vision, moderate impairment	15	0.394	0.252	0.505
Hearing loss, complete	13	0.405	0.282	0.479
Hearing loss, moderate	22	0.357	0.240	0.437
Musculoskeletal disorders				
Low back pain, chronic, with leg pain	11	0.409	0.288	0.479
Musculoskeletal problems, legs, severe	29	0.310	0.220	0.363
Musculoskeletal problems, legs, moderate	28	0.314	0.211	0.388
Musculoskeletal problems, arms, moderate	43	0.214	0.135	0.285
Injuries				
Traumatic brain injury, long-term consequences, severe, with or without treatment	2	0.536	0.371	0.632
Spinal cord lesion at neck, treated	30	0.306	0.197	0.394
Amputation of one leg, long term, without treatment	3	0.504	0.330	0.626
Amputation of one leg, long term, with treatment	45	0.200	0.131	0.259
Amputation of one arm, long term, with or without treatment	38	0.254	0.165	0.326
Amputation of both arms, long term, with treatment	49	0.058	0.029	0.101
Amputation of finger(s), excluding thumb, long term, with treatment	39	0.252	0.168	0.317
Dislocation of shoulder, long term, with or without treatment	21	0.360	0.241	0.443
Fracture of neck of femur, long term, with treatment	25	0.339	0.219	0.431
Fracture of radius or ulna, long term, without treatment	4	0.488	0.332	0.585
Severe chest injury, long term, with or without treatment	36	0.265	0.176	0.334
Burns of <20% total surface area or <10% total surface area if head or neck, or hands or wrist involved, long term, with or without treatment	6	0.484	0.314	0.608
Other				
Disfigurement, level 3	17	0.371	0.258	0.440
Disfigurement, level 1	26	0.325	0.200	0.433
Itching or pain	40	0.248	0.158	0.325
Abdominopelvic problem, severe	27	0.319	0.217	0.458
Severe tooth loss	44	0.209	0.140	0.264
Periodontitis	46	0.175	0.102	0.250
Anaemia, moderate	33	0.298	0.189	0.388

CI: confidence interval, LL: lower limit, UL: upper limit.

**Fig. 1:**
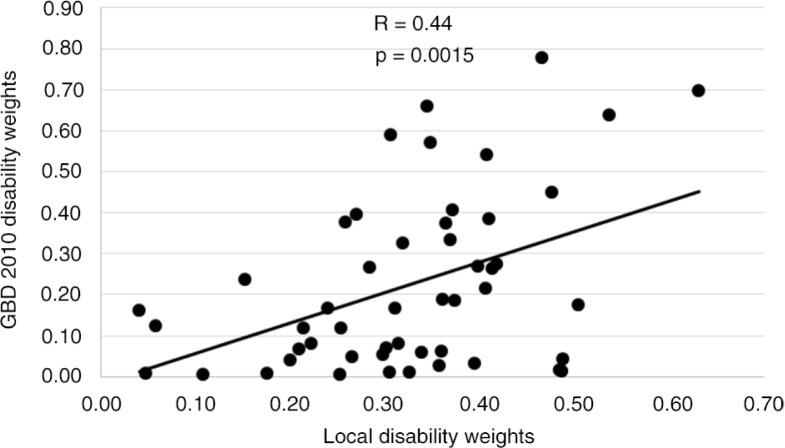
Correlation of health state disability weights between the local and GBD 2010 studies. R represents the Pearson's correlation coefficient.

The Pearson's correlation coefficient between the disability weights of the local study and those of the GBD study was 0.44 (*p*=0.0015; Fig. 1). The correlations between the disability weights within each health state domain were poor except for the mental, behavioural, and substance-use domain which had a Pearson's correlation coefficient of 0.66 (*p*>0.05) and the musculoskeletal disorders domain (*r*=0.79, *p*>0.05). The point estimates of the local study were within the 95% confidence interval of the GBD 2010 study for 25.5% of all health states, whereas 58.8% were higher than the upper bound and 15.7% were below the lower bound of the GBD 2010 uncertainty interval.

## Discussion

This is the first study to our knowledge that has attempted to derive disability weights for a range of health states from the general public living in a resource-deprived community in South Africa. The health states with the highest and lowest disability weights seem plausible; however, there were also some counterintuitive results. The overall correlation of the disability weights between the local and GBD 2010 study was moderate but statistically significant, indicating a relationship between the disability weights of the two studies. However, there was considerable variability in the disability weights for selected conditions, such as ear pain (0.486 in local study and 0.013 in GBD 2010), untreated fracture of the radius or ulna (0.488 in local study and 0.043 in GBD 2010), and amputation of one leg, untreated (0.504 in local study and 0.173 in GBD 2010). The correlation coefficient would have been stronger if the ranking of certain health states was not as counterintuitive. For instance, moderate dementia ranked lower than mild dementia, severe alcohol-use disorder ranked lower than moderate-use disorder, and blindness ranked lower than both severe and moderate vision impairment. The GBD assertion of universality of health state preferences is not entirely supported by the results of this study. This finding therefore raises questions about circumstantial factors that may influence perceptions of health states.

Untreated injuries ranked particularly high, whereas severe intellectual disability ranked lowest, which might indicate that local respondents value physical mobility higher than cognitive functioning. In the GBD 2010 study, mental disorders such as acute schizophrenia (disability weight 0.776) and severe major depression (disability weight 0.658) had among the highest disability weights, whereas injuries such as amputation of one leg without treatment ranked much lower with a disability weight of 0.173. These differences might be contextual because the South African research site is an impoverished community whereas most respondents in the GBD 2010 were from the United States and Australia, and had tertiary education and high living standards. There have been other studies which suggest that contextual factors influence differing health state preferences. In a review of disability weight studies, Haagsma et al. (2) indicated that even when fairly similar methodological valuation designs were used, there were marked differences in disability weights for the same health state between different studies. For instance, Stouthard et al. (15) and Lai et al. (16) reported disability weights for severe depression of 0.147 and 0.83, respectively. Both studies used medical experts as study respondents, a disease-specific health state description (Stouthard et al. (15) added an EQ-5D component to the disease-specific health state description), the person trade-off valuation method to valuate indicator conditions, and the visual analogue scale to assess the other conditions. The difference in outcome in these two studies may point to contextual differences, which is possible because the Stouthard et al. (15) study was done in the Netherlands whereas the Lai et al. (16) study was done in Estonia. The understanding of the symptoms and consequences of ill health relayed by health state descriptions may be shaped by cultural and social values, and does not remain constant because of external changes over time (17). A health state valuation may depend on the social stigma or disruption to social life for one person, whereas another might valuate the same health state on the basis of loss of working ability or time loss (17).

The wording of the health state descriptions might be important in the understanding of the severity of health states (12). The descriptions for health states within the mental, behavioural, and substance-use disorders domain included the cause of the health state, for example, ‘drinking of alcohol’, whereas this was excluded from most other health state descriptions. This could have made it easier for participants to relate to the health state, which might explain the good correlation of health states within this domain.

The overall approach used to valuate health states may also not be effective in producing reliable results in all settings. The techniques used in valuating health states can be cognitively demanding (18) with respondents usually not familiar with the method, and the design often does not allow enough time to reflect on the health state choices made (19). In addition, the task, which involves making multiple complex choices, is quite strenuous (17). The counterintuitive results observed in the local study, such as severe and moderate vision impairment ranking higher than blindness, might suggest that the health state descriptions were not well understood. There was no repetition of the first and last paired comparison questions, which could have given an indication of whether the methodology was well understood by the respondents.

The GBD study group recently published new disability weights for their 2013 analysis of the global disease burden (20). Changes have been made to some health state descriptions to add consistency in wording and additional health states have been added. The study pooled the GBD 2010 disability weights with disability weights from a study conducted in five European countries (14). The health states that were the same in the GBD 2010 and GBD 2013 study showed a high degree of correlation with a Pearson's correlation coefficient of 0.992. The GBD 2013 study involved double the number of respondents to the 2010 study but they were mostly from high-income countries. Hence, more studies from low-resource settings are needed to test the GBD assertion regarding the universality of their disability weights.

### Limitations

A possible limitation of this study is that the survey questionnaire was presented in English, whereas the home language of some participants was Afrikaans. However, during pilot testing respondents showed good understanding of the questions and the ability to reason rationally. The health state rankings may also have been influenced by the sample size which may not have allowed for all possible pairs of health states to be sufficiently compared in the pairwise comparison health state valuation. Another limitation is that only one TTO exercise was assigned to each respondent. The cognitive difficulty of a TTO might require more than one exercise before an understanding of the concept is developed.

## Conclusion

In conclusion, this study is unable to refute the claim that health state preferences are universal although it does show differences in the preference of health states between the local and GBD study. A universal set of disability weights might be preferable for comparing DALYs between countries; however, the counter argument is that empirical disability weights are needed to better represent DALY estimates for each country. Although country-specific disability weights would be ideal, the current methods used to assess health state preferences by the GBD group might not be feasible in all settings. To derive empirical disability weights in low socio-economic settings might require methods that are less cognitively demanding for respondents. A visual analogue scale might be the easiest health valuation method to explain to respondents. However, validation studies that test for the best methods in such settings are advisable.

The DALY is a valuable tool as it gives a comprehensive picture of morbidity and mortality for different diseases using a single metric allowing easier decisions regarding resource allocation towards diseases with high burden (21, 22). However, accurate estimates of mortality and morbidity are also needed in addition to disability weights, especially in resource-constrained countries such as South Africa.

## Paper context

Health state disability weights are universally applied by the GBD study to estimate DALYs for each country, despite social and cultural differences. This paper suggests that the GBD disability weights for some health states are not necessarily representative of a economically disadvantaged community and the methods used to valuate health states might not be suitable for all settings. Studies estimating DALYs need to derive local disability weights using less cognitively demanding methods.
